# Operative versus Nonoperative Treatment in Patients with Advanced Non-Small-Cell Lung Cancer: Recommended for Surgery

**DOI:** 10.1155/2023/4119541

**Published:** 2023-01-12

**Authors:** Hui Wang, Di Yang, Yan Lv, Jing Lin, Haibin Wang

**Affiliations:** ^1^Department of Clinical Laboratory, The Fourth Medical Centre, Chinese PLA General Hospital, No. 51 Fucheng Road, Beijing 100037, China; ^2^Department of Orthopedics Surgery, Chinese PLA General Hospital, No. 51 Fucheng Road, Beijing 100037, China; ^3^Army Medical University NCO School, 450 Zhongshan Xi Lu, Shijiazhuang 050083, Hebei, China; ^4^Department of Respiratory and Critical Care Medicine, The Fourth Medical Centre, Chinese PLA General Hospital, No. 51 Fucheng Road, Beijing 100037, China

## Abstract

**Background:**

There is currently limited evidence for a correlation between the recommended operation and overall survival (OS) in patients with advanced non-small-cell lung cancer (NSCLC).

**Methods:**

NSCLC patients with stages III and IV, recommended for operation, were identified in the US National Cancer Institute Surveillance, Epidemiology, and End Results database (SEER).We used propensity score matching (PSM) and multivariable Cox proportional hazards regression to ensure the robustness of our findings. The cumulative rates of death were compared between patients with and without recommended operations using the Kaplan−Meier curves.

**Results:**

Operation was recommended for 3331 patients but was not performed in 912 (27.4%) patients (then on-operative group). After PSM, 553 pairs matched. Compared to the nonoperative group, the hazard ratios (HRs) in the operative group were 0.46 (95% CI 0.23–0.95 and *p*=0.037) in stage IIIA and 0.54 (95% CI 0.42–0.68 and *p* < 0.001) in stage IVA. However, in stages IIIB, IIIC, and IVB, the recommended operative group was not associated with better OS. The OS was not different in stage IIIA-N2, stage IVA-N1, and stage IVA-N3 patients between groups (*p*=0.28, *p*=0.14, and *p*=0.79, respectively). Moreover, the recommended operative group had better OS than the nonoperative group in stage IIIA-N0 (*p*=0.00085), stage IIIA-N1 (*p*=0.009), stage IVA-N0 (*p* < 0.001), and stage IVA-N2 (*p*=0.034).

**Conclusion:**

Compared to the nonoperative group, recommended operation improved survival in NSCLC patients with stage IIIA-N0, stage IIIA-N1, stage IVA-N0, and stage IVA-N2. However, in stages IIIA-N2, IIIB, IIIC, IVA-N1, IVA-N3, and IVB, recommended operation did not lead to significantly improved survival time.

## 1. Introduction

Lung cancer is the world's leading cause of cancer death [1–3]. Nearly 80% of all lung cancer patients are diagnosed with non-small-cell lung cancer (NSCLC) [3, 4]. In clinical practice, approximately 75% of patients already have an advanced stage of NSCLC at the time of diagnosis [5, 6]. Despite significant improvements in the treatment of advanced NSCLC in recent years, survival remains poor, with a five-year survival rate below 6% [3, 7]. The role of operation as one part of multimodality management for advanced-stage patients is persistent but controversial [8, 9].

As the primary local therapy approach, the oncologist performed operative resection of the primary tumor in selected patients with advanced NSCLC [10]. A series of small retrospective research studies provided contradicting results on the benefits of operations [11–13]. Randomized controlled trials suggested that operative resection may not enhance overall or progression-free survival in NSCLC patients with stage IIIA-N2 [14, 15], noting that one of the trials only included patients with unresectable tumors. Previous studies have mainly focused on operative treatment for stage I–IIIA NSCLC and provided less operative information on more advanced NSCLC [16, 17]. Moreover, according to the NCCN guidelines, doctors should consider aggressive local therapy for patients with limited metastases in the context of multimodality treatment [18–21]. However, there is little basis for physicians to make robust judgments about the appropriate treatment strategies and protocols for NSCLC patients [22, 23]. Hence, retrospective cohort research based on real-world populations may be valuable for clinicians to identify operative candidates who are likely to have improved survival outcomes and thus further support better treatment decisions.

Therefore, we conducted a large-scale retrospective cohort study through the US National Cancer Institute Surveillance, Epidemiology, and End Results (SEER) database. The primary purpose was to investigate the relationship between operative treatment and overall survival (OS) in advanced NSCLC patients. The second purpose was to determine the clinical characteristics of patients associated with overall survival benefits from the operation, consisting of patient information at the time of the initial diagnosis of NSCLC.

## 2. Methods

### 2.1. Data

This cohort research adopted the SEER database (the November 2021 submission). We obtained the patient database through SEER*∗*Stat software (SEER Stat 8.4.0).

### 2.2. Inclusion/Exclusion Criteria

Patients diagnosed with NSCLC (malignant neoplasm of the lung and bronchus, NSCLC histology, and one primary) between 2010 and 2019 were recruited from the database.

Inclusion criteria were (1) patients with pathologically confirmed NSCLC and recommended for surgery, (2) those with stage III and IV following the 8th edition of the American Joint Committee on Cancer (AJCC) TNM classification, and (3) those who were diagnosed as the first primary malignancy.

Exclusion criteria included (1) patients diagnosed with other histological types (e.g., small cell lung cancer, ICD-0-3 8041–8045) and (2) patients with incomplete data (e.g., incomplete survival months, unknown primary tumor location, or unspecified diagnostic confirmation). The data and codes were documented by the North American Association of Central Cancer Registries. The site and histology of primary cancer were coded using the International Classification of Diseases (ICD-O-3). The Research Ethics Board of the Chinese PLA General Hospital exempted the study from ethical approval because the author could not get the patient's identity information. We obtained data agreement according to the requirements of the SEER database.

### 2.3. Variables

Operation treatment was defined as a record of the following: (1) surgery performed or (2) recommended but not performed in “Reason no cancer-directed surgery” in the SEER database.

### 2.4. Covariates

According to the published guidelines and research, we obtained the following variables: (1) demographic information, (2) variables that could affect cancer-directed operation for NSCLC or OS reported by previous literature, and (3) other relevant information on account of clinical experience. The following variables were adopted to construct the adjusted models: age, sex, race, marital status, primary site, grade, laterality, histology, AJCC stage, radiation, chemotherapy, and type of surgery.

### 2.5. Outcomes

The outcome was OS. Since the date is a confidential variable in some US registries, a process was established so that a SAS code could be downloaded from the data site. A registry can run the SAS code locally and provide the length of survival in the month for analysis.

### 2.6. Statistical Analysis

All skewed or normally distributed continuous data were presented as median (Mdn) and interquartile range (IQR) or mean ± standard deviation (SD) as appropriate. Categorical data were expressed in frequency or as percentages. We compared the characteristics of the operative group with the nonoperative group using *t*-tests (normal distribution), Mann−Whitney tests (skewed distribution), or *χ*^2^ tests (categorical variables), where appropriate.

This article followed the Strengthening the Reporting of Observational Studies in Epidemiology (STROBE) Statement. No imputation was performed because the percentage of missing data was small (0–6%). We used propensity score-matched analysis (PSM) to minimize baseline differences. Baseline matching variables consisted of age, sex, race, marital status, primary site, laterality, histology, AJCC stage, radiation, and chemotherapy. We paired the nonoperative group and the operative group using exact matching with a caliper size of 0.2 based on the propensity scores. The cumulative rates of death were compared using the Kaplan−Meier curves.

We established multivariate Cox proportional hazard models to investigate the factors associated with overall survival. We assessed the associations between operative treatment and OS using hazard ratios (HRs) and 95% confidence intervals (CIs). Due to the differences in distribution between the groups, the adjusted Cox proportional hazards models included age, sex, race, marital status, primary site, grade, laterality, histology, AJCC stage, radiation, chemotherapy, and type of surgery.

The statistical software package R version 4.0.2 (R Foundation for Statistical Computing, Vienna, Austria) and free statistics software version 1.3 performed all statistical analyses. A *p* value < 0.05 (two-sided) was statistically significant.

## 3. Results

### 3.1. Baseline Characteristics of NSCLC Patients

**Figure 1:**
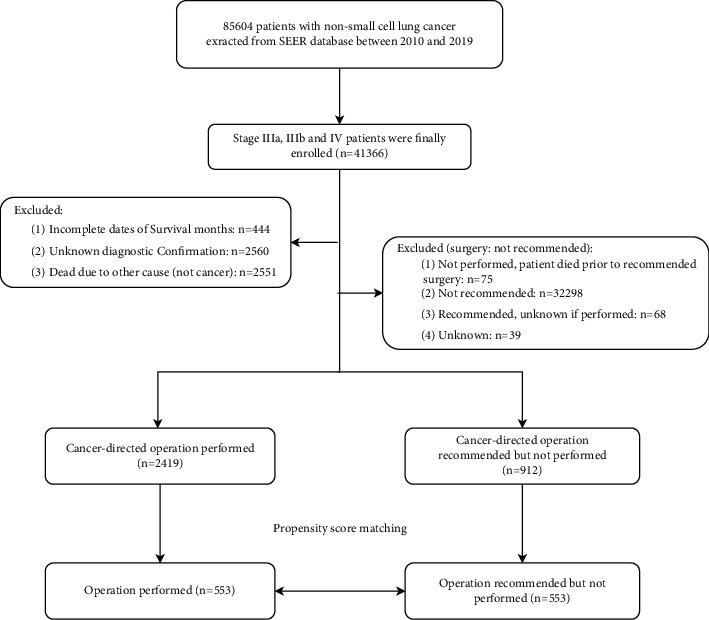
The flow chart of the study.

A total of 3331 patients with NSCLC who were recommended for surgery were enrolled in this research. Of these patients, 912 (27.4%) were recommended for surgery but not performed (the nonoperative group), and the remainder underwent surgery (the operative group). After propensity score-matched analysis, patient characteristics were balanced across groups, and 1106 patients with NSCLC who were recommended for the operation were enrolled in this research (Figure 1). The demographic and clinical characteristics of NSCLC patients before and after PSM are summarized in Table 1. Additionally, 3331 advanced NSCLC patients who were recommended for surgery were alive or dead due to cancer in this study.

After PSM, 553 pairs matched (Supplementary Figure 1). The mean age was 69.0 ± 10.7 years; 493 (44.6%) were women, and 897 (81.1%) were Caucasian. The numbers of patients in each stage were 201, 120, and 20 for stages IIIA, IIIB, and IIIC; 725 and 40 for stages IVA and IVB, respectively.

### 3.2. Cox Proportional Hazard Regression Analysis

We constructed multivariate models to evaluate the associations between the operation and OS (Table 2). The operation was independently associated with improved OS in the entire advanced cohort (Model 3: HR = 0.57, 95% CI 0.47–0.69, and *p* < 0.001). Compared to the nonoperative group, the hazard ratios (HRs) in the operative group were 0.46 (95% CI 0.23–0.95 and *p*=0.037) in stage IIIA and 0.54 (95% CI 0.42–0.68 and *p* < 0.001) in stage IVA. However, the recommended operation was not independently associated with improved OS in stages IIIB, IIIC, and IVB. Moreover, HRs were similar between the unadjusted model and the adjusted models with imputed covariate data.

After adjustment for all covariates, in stage IIIA, compared to the reference group, Grade II (HR = 6.2, 95% CI 2.09–18.4, and *p*=0.001) and Grade III (HR = 4.68, 95% CI 1.51–14.5, and *p*=0.008) were shown to be risk predictors of OS, and adenomas and adenocarcinomas (HR = 0.12, 95% CI 0.04–0.36, and *p* < 0.001) were associated with a reduced hazard for OS (Table 3). In stage IVA, age (HR = 1.02, 95% CI 1.01–1.03, and *p* < 0.001) was an independent predictor of OS. The survival benefit might be most prominent with Asian or Pacific Islanders (HR = 0.62, 95% CI 0.4∼0.95, and *p*=0.028) and chemotherapy (HR = 0.52, 95% CI 0.39–0.69, and *p* < 0.001) (Table 4). Moreover, in both stages IIIA and IVA, the female was shown to be a significant beneficial predictor of survival in stage IIIA: HR = 0.37, 95% CI 0.17–0.82, and *p*=0.014 and in stage IVA: HR = 0.77, 95% CI 0.61–0.99, and *p*=0.039 (Tables 3 and 4).

### 3.3. Survival Analysis

**Figure 2:**
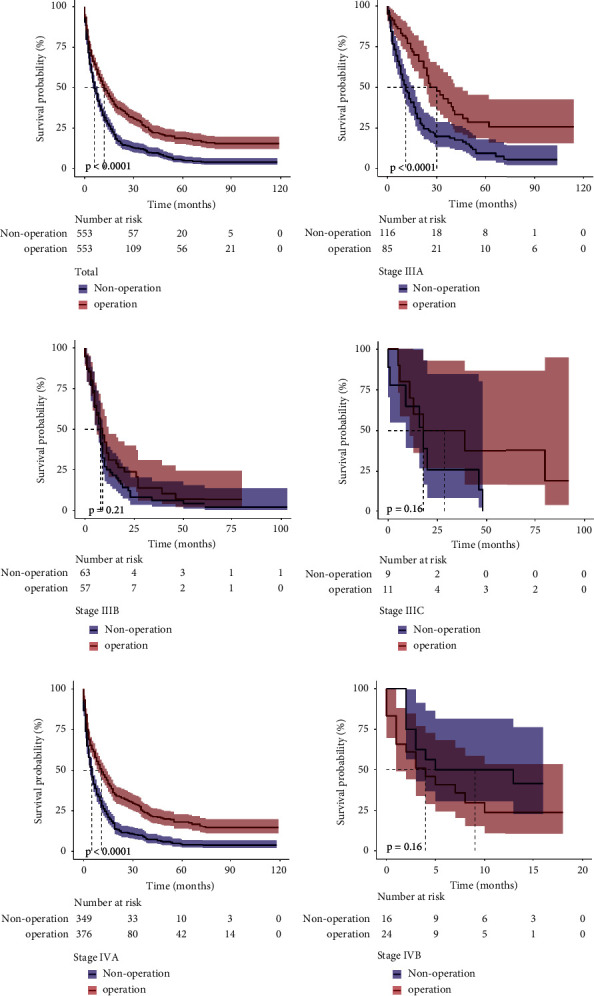
Kaplan−Meier plots for overall survival. (a) Entire cohort; (b) stage IIIA; (c) stage IIIB; (d) stage IIIC; (e) stage IVA; (f) stage IVB.

We conducted Kaplan−Meier analyses to compare the survival rates of the operative and nonoperative groups. The operative patients had better OS than the nonoperative patients in stages IIIA and IVA (*p* < 0.001, Figure 2). However, the improved OS was not significantly different between the operative and nonoperative groups in stage IIIB, IIIC, and IVB patients (*p*=0.21, *p*=0.16, and *p*=0.16, respectively, Figure 2).

**Figure 3:**
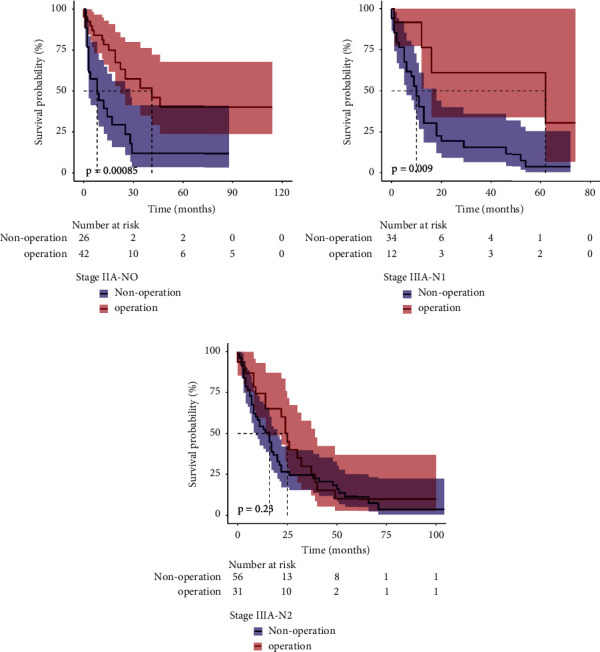
Kaplan−Meier plots for overall survival. (a) Stage IIIA-N0; (b) stage IIIA-N1; (c) stage IIIA-N2.

**Figure 4:**
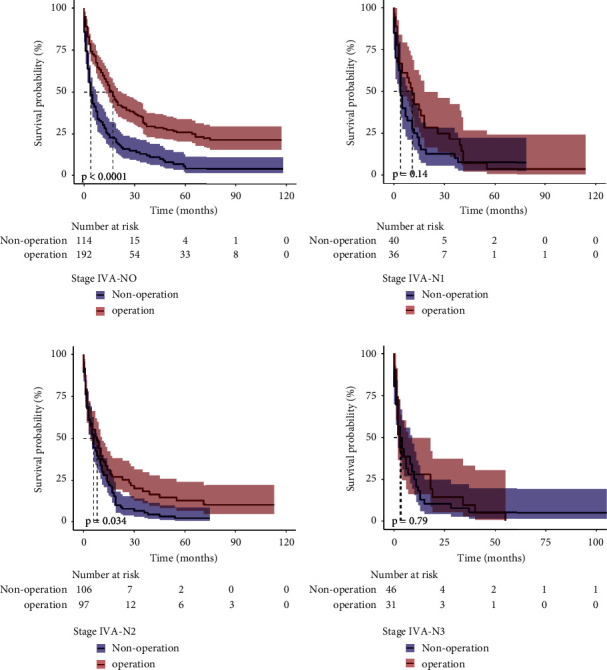
Kaplan−Meier plots for overall survival. (a) Stage IVA-N0; (b) stage IVA-N1; (c) stage IVA-N2; (d) stage IVA-N3.

We evaluated several secondary outcomes to investigate potential factors that might have contributed to the benefits of the operation in stages IIIA and IVA. The operative patients had better OS than the nonoperative patients in stages IIIA-N0 and N1 (*p*=0.00085 and *p*=0.009, respectively); however, there was no difference in stage IIIA-N2 patients between the groups (*p*=0.28) (Figure 3). In stage IVA-N0 and N2, the operative group had better OS than the nonoperative group (*p* < 0.001 and *p*=0.034, respectively); however, there was no difference in stage IVA-N1 and N3 patients between the groups (*p*=0.14 and *p*=0.79, respectively) (Figure 4).

## 4. Discussion

Recently, for patients with advanced-stage NSCLC, chemotherapy and radiation therapy have been the primary management modalities [24], and the role of recommended operative treatment remains ambiguous. Our study found that recommended operative treatment was not significantly associated with improved survival outcomes in NSCLC patients with stages IIIB, IIIC, and IVB. In addition, our results showed that recommended operative treatment appeared to result in improved survival for NSCLC patients with stages IIIA-N0, IIIA-N1, IVA-N0, and IVA-N2. Our findings will provide the basis for clinicians to select which kinds of advanced NSCLC patients would benefit from recommended operative treatment.

Among patients with stages IIIA-N2, IIIB, and IIIC, we found that recommended surgery did not significantly enhance overall survival, but patients with stages IIIA-N0 and N1 did. As mentioned previously, randomized controlled trials suggested that in the selected stage IIIA-N2 patients with responses to induction chemotherapy, the effect of operative treatment on overall or progression-free survival was not significantly better than radiotherapy [14]. In view of this, clinicians might adopt radiotherapy as the preferred choice of local treatment for these advanced NSCLC patients [14, 15]. Their conclusions are consistent with our findings, and our research further strengthens the previous results. However, the abundance of this patient cohort allowed us to further investigate the potential benefits of having recommended the surgery in appropriately selected NSCLC patients with stage III.

The latest treatment guidelines have recommended operative resection in selected NSCLC patients with stage IV, such as those cases with early-stage lung cancer and limited extrathoracic metastatic tumors, but there was minimal evidence [19]. Our result proved that stage IVB NSCLC patients who undergo the recommended surgery are unlikely to have better OS than the nonoperative group, but those with stages IVA-N0 and IVA-N2 might have a better OS. Previous studies have shown that surgery for cT1-2, N0-1, M1 or cT3, N0, and M1 disease did not seem to affect prognosis when compared with nonoperative therapy; however, because surgery does not provide an obvious benefit, they should not be recommended to stage IV patients with mediastinal nodal cases or more locally advanced tumors [13]. These conclusions are inconsistent with our findings. However, some other studies were consistent with our results. Yamaguchi et al. [12] reported that by utilizing the local treatment for distant metastases and therapeutic pneumonectomy, some M1b-cStage IV NSCLC patients had more prolonged survival than others. Kawano et al. [11] reported that operative treatment could prolong the survival of NSCLC patients with stage IV on the premise that patients can tolerate surgery. In the clinical setting, stage IV NSCLC patients rarely undergo curative-intent resections. However, a strength of our study was that we were able to use a robust statistical method and an extensive comprehensive population-based SEER database to determine the OS gain among NSCLC patients. After controlling for age, sex, race, marital status, primary site, grade, laterality, histology, AJCC stage, radiation, chemotherapy, and type of surgery. We found that stage IVA patients with chemotherapy might have a significantly better impact on the survival benefits. This finding implied that chemotherapy might have a more positive effect in determining the survival benefits of stage IV patients.

A logistic univariate analysis revealed the following possible reasons for refusal to recommend surgery for patients with advanced NSCLC: stage at diagnosis, age, gender, race, marital status, and histology. Surgery is the preferred treatment for stage I lung cancer since it is curable. There are other powerful and healing therapies available. However, some patients only receive palliative treatment, while others get no treatment at all [25]. According to the search [25], comorbidities, patient preferences, and illness progression are the main causes of treatment refusal. Therefore, in addition to the previously mentioned reasons in this study, the impact of comorbidities, patient decisions, disease progression, patients' financial ability, and acceptance of doctors and techniques on whether advanced NSCLC patients refuse recommended surgery is thus poorly understood and requires further research.

Primary treatment strategies might be changing as systemic therapies develop, but some researchers have suggested that positive responses to systemic therapy would make more NSCLC patients eligible for operative management [18, 26, 27]. According to the Robinson classification, specific cases of N2-patients had a higher possibility of survival as a part of multimodal therapy after an operative therapy [28, 29]. Immunotherapies have revolutionized the treatment of advanced NSCLC. Evidence has emerged that it can be used for stage III disease [30, 31]. However, how best to combine surgery with other new therapies needs more studies. In addition, for stage III and IV patients, further studies are needed to find better therapeutic options for NSCLC patients.

Nevertheless, this study has several limitations. Firstly, our analysis is based on the assumption that clinicians followed consistent criteria (e.g., NCCN guidelines) to recommend the most appropriate treatments to patients. Secondly, our study was powerless to assess the effect of other therapies (e.g., the multimodality approach) on survival when combined with resection surgery. Although we recruited 3331 advanced NSCLC patients who were recommended for surgery, all of whom were alive or dead due to cancer, we were unable to assess the impact of comorbidities on outcomes. Thirdly, although recommended primary tumor resection might not have an impact on the survival benefits in stage IVA-N1 and N3 patients, the sample size was limited. Finally, this article relies heavily on statistical analysis, and we must acknowledge the limitations of statistics. In future studies, a larger sample size would be preferred to validate the findings shown in this study. Besides, we would like to emphasize that correlation does not imply causation. Further study is required to understand the direct cause of improved OS in the specific advanced NSCLC patients after recommended resection surgery.

In conclusion, this NSCLC population-based study found that recommended operation was associated with prolonged survival in stage IIIA-N0, stage IIIA-N1, stage IVA-N0, and stage IVA-N2 patients compared with those who were recommended for operation but not performed. However, in NSCLC patients with stages IIIA-N2, IIIB, IIIC, IVA-N1, IVA-N3, and IVB, no evidence suggests that the recommended operation could significantly improve survival time.

## Figures and Tables

**Table 1:** Baseline clinical and demographic characteristics of advanced NSCLC patients.

Characteristic	Surgery recommended (unmatched)				Surgery recommended (propensity-score-matched)			
	Total (*n* = 3331)	Nonoperation (*n* = 912)	Operation (*n* = 2419)	*p* value	Total (*n* = 1106)	Nonoperation (*n* = 553)	Operation (*n* = 553)	*p* value
Age (SD, years)	66.6 ± 11.3	71.4 ± 11.4	64.8 ± 10.8	<0.001	69.0 ± 10.7	69.5 ± 10.9	68.6 ± 10.5	0.183
Age <= 30, *n* (%)	11 (0.3)	0 (0)	11 (0.5)	<0.001	2 (0.2)	0 (0)	2 (0.4)	0.098
30 < age <= 60, *n* (%)	847 (25.4)	143 (15.7)	704 (29.1)		209 (18.9)	109 (19.7)	100 (18.1)	
60 < age <= 90, *n* (%)	2434 (73.1)	732 (80.3)	1702 (70.4)		888 (80.3)	438 (79.2)	450 (81.4)	
Age > 90, *n* (%)	39 (1.2)	37 (4.1)	2 (0.1)		7 (0.6)	6 (1.1)	1 (0.2)	
Sex, *n* (%)				0.036				0.304
Male	1717 (51.5)	497 (54.5)	1220 (50.4)		613 (55.4)	315 (57)	298 (53.9)	
Female	1614 (48.5)	415 (45.5)	1199 (49.6)		493 (44.6)	238 (43)	255 (46.1)	
Race, *n* (%)				0.034				0.886
Caucasian	2651 (79.8)	740 (81.3)	1911 (79.3)		897 (81.1)	454 (82.1)	443 (80.1)	
African American	269 (8.1)	56 (6.2)	213 (8.8)		90 (8.1)	42 (7.6)	48 (8.7)	
American Indian/Alaska native	24 (0.7)	10 (1.1)	14 (0.6)		6 (0.5)	3 (0.5)	3 (0.5)	
Asian or Pacific Islander	377 (11.4)	104 (11.4)	273 (11.3)		113 (10.2)	54 (9.8)	59 (10.7)	
Marital status at diagnosis, *n* (%)				<0.001				0.946
Single	480 (15.1)	147 (17.7)	333 (14.2)		179 (16.2)	91 (16.5)	88 (15.9)	
Unmarried or domestic partner	21 (0.7)	2 (0.2)	19 (0.8)		6 (0.5)	2 (0.4)	4 (0.7)	
Married	1818 (57.4)	392 (47.3)	1426 (60.9)		582 (52.6)	290 (52.4)	292 (52.8)	
Separated	26 (0.8)	8 (1)	18 (0.8)		10 (0.9)	6 (1.1)	4 (0.7)	
Divorced	407 (12.8)	98 (11.8)	309 (13.2)		142 (12.8)	69 (12.5)	73 (13.2)	
Widowed	418 (13.2)	182 (22)	236 (10.1)		187 (16.9)	95 (17.2)	92 (16.6)	
Primary site, *n* (%)				<0.001				0.075
Main bronchus	110 (3.3)	27 (3)	83 (3.4)		58 (5.2)	18 (3.3)	40 (7.2)	
Upper lobe and lung	1653 (49.6)	400 (43.9)	1253 (51.8)		526 (47.6)	277 (50.1)	249 (45)	
Middle lobe and lung	166 (5.0)	41 (4.5)	125 (5.2)		62 (5.6)	31 (5.6)	31 (5.6)	
Lower lobe and lung	957 (28.7)	204 (22.4)	753 (31.1)		289 (26.1)	144 (26)	145 (26.2)	
Overlapping lession of lung	66 (2.0)	6 (0.7)	60 (2.5)		10 (0.9)	5 (0.9)	5 (0.9)	
Grade, *n* (%)				0.039				0.585
I: well differentiated	159 (8.4)	29 (8.8)	130 (8.4)		53 (9.6)	23 (9.6)	30 (9.6)	
II: moderately differentiated	722 (38.3)	103 (31.4)	619 (39.8)		189 (34.2)	88 (36.7)	101 (32.3)	
III: poorly differentiated	950 (50.5)	185 (56.4)	765 (49.2)		287 (51.9)	121 (50.4)	166 (53)	
IV: undifferentiated anaplastic	52 (2.8)	11 (3.4)	41 (2.6)		24 (4.3)	8 (3.3)	16 (5.1)	
Laterality, *n* (%)				<0.001				0.972
Right	1845 (55.7)	465 (52.1)	1380 (57.1)		641 (58.0)	319 (57.7)	322 (58.2)	
Left	1354 (40.9)	334 (37.4)	1020 (42.2)		436 (39.4)	219 (39.6)	217 (39.2)	
Bilateral involvement	111 (3.4)	93 (10.4)	18 (0.7)		29 (2.6)	15 (2.7)	14 (2.5)	
Histology, *n* (%)				<0.001				0.842
Epithelial neoplasms	421 (12.6)	248 (27.2)	173 (7.2)		168 (15.2)	86 (15.6)	82 (14.8)	
Squamous cell neoplasms	733 (22.0)	199 (21.8)	534 (22.1)		270 (24.4)	141 (25.5)	129 (23.3)	
Adenomas and adenocarcinomas	1833 (55.0)	444 (48.7)	1389 (57.4)		628 (56.8)	308 (55.7)	320 (57.9)	
Acinar cell neoplasms	192 (5.8)	3 (0.3)	189 (7.8)		8 (0.7)	3 (0.5)	5 (0.9)	
Others†	152 (4.6)	18 (2)	134 (5.5)		32 (2.9)	15 (2.7)	17 (3.1)	

Type of surgery*∗*	912 (27.7)	912 (100)	0 (0)	<0.001				<0.001
Local tumor destruction	98 (3.0)	0 (0)	98 (4.1)		94 (8.6)	0 (0)	94 (17.4)	
Resection of less than one lobe	541 (16.4)	0 (0)	541 (22.7)		328 (30.0)	0 (0)	328 (60.6)	
Lobectomy	1361 (41.3)	0 (0)	1361 (57.2)		102 (9.3)	0 (0)	102 (18.9)	
Lobe or bilobectomy extended	118 (3.6)	0 (0)	118 (5)		8 (0.7)	0 (0)	8 (1.5)	
Pneumonectomy	251 (7.6)	0 (0)	251 (10.5)		7 (0.6)	0 (0)	7 (1.3)	
Extended pneumonectomy	11 (0.3)	0 (0)	11 (0.5)		2 (0.2)	0 (0)	2 (0.4)	
T, *n* (%)				0.052				0.003
T1a	147 (4.8)	24 (3.5)	123 (5.2)		47 (4.8)	18 (3.8)	29 (5.6)	
T1b	190 (6.2)	47 (6.8)	143 (6.1)		56 (5.7)	30 (6.4)	26 (5)	
T1c	47 (1.5)	7 (1)	40 (1.7)		12 (1.2)	7 (1.5)	5 (1)	
T2a	591 (19.4)	118 (17)	473 (20.1)		174 (17.6)	84 (17.8)	90 (17.5)	
T2b	190 (6.2)	51 (7.3)	139 (5.9)		54 (5.5)	38 (8.1)	16 (3.1)	
T3	909 (29.8)	205 (29.5)	704 (29.9)		274 (27.8)	139 (29.4)	135 (26.2)	
T4	976 (32.0)	243 (35)	733 (31.1)		370 (37.5)	156 (33.1)	214 (41.6)	
N, *n* (%)				<0.001				<0.001
N0	826 (26.2)	239 (31.2)	587 (24.6)		384 (37.0)	144 (28.3)	240 (45.2)	
N1	592 (18.8)	109 (14.2)	483 (20.3)		124 (11.9)	76 (15)	48 (9)	
N2	1531 (48.6)	316 (41.3)	1215 (51)		414 (39.8)	224 (44.1)	190 (35.8)	
N3	201 (6.4)	102 (13.3)	99 (4.2)		117 (11.3)	64 (12.6)	53 (10)	
M, *n* (%)				<0.001				<0.001
M0	1871 (57.4)	217 (25)	1654 (69.1)		341 (32.0)	188 (35.6)	153 (28.5)	
M1a	533 (16.3)	227 (26.2)	306 (12.8)		317 (29.8)	118 (22.3)	199 (37.1)	
M1b	790 (24.2)	400 (46.1)	390 (16.3)		367 (34.5)	206 (39)	161 (30)	
M1c	66 (2.0)	23 (2.7)	43 (1.8)		40 (3.8)	16 (3)	24 (4.5)	
AJCC stage, *n* (%)				<0.001				0.096
Stage IIIA	1359 (40.8)	127 (13.9)	1232 (50.9)		201 (18.2)	116 (21)	85 (15.4)	
Stage IIIB	473 (14.2)	70 (7.7)	403 (16.7)		120 (10.8)	63 (11.4)	57 (10.3)	
Stage IIIC	39 (1.2)	20 (2.2)	19 (0.8)		20 (1.8)	9 (1.6)	11 (2)	
Stage IVA	1394 (41.8)	672 (73.7)	722 (29.8)		725 (65.6)	349 (63.1)	376 (68)	
Stage IVB	66 (2.0)	23 (2.5)	43 (1.8)		40 (3.6)	16 (2.9)	24 (4.3)	
Radiation, *n* (%)				<0.001				0.229
No/unknown	2101 (63.1)	685 (75.1)	1416 (58.5)		725 (65.6)	372 (67.3)	353 (63.8)	
Yes	1230 (36.9)	227 (24.9)	1003 (41.5)		381 (34.4)	181 (32.7)	200 (36.2)	
Chemotherapy, *n* (%)				<0.001				0.298
No/unknown	1407 (42.2)	660 (72.4)	747 (30.9)		655 (59.2)	336 (60.8)	319 (57.7)	
Yes	1924 (57.8)	252 (27.6)	1672 (69.1)		451 (40.8)	217 (39.2)	234 (42.3)	
Tumor size and mean ± SD (mm)	48.2 ± 31.7	48.8 ± 27.1	48.1 ± 32.2	0.858	47.8 ± 31.8	49.0 ± 27.5	47.3 ± 33.5	0.712

†, histology and other types included: transitional cell papillomas and carcinomas (*n* = 1), mucoepidermoid neoplasms (*n* = 5), cystic, mucinous, and serous neoplasms (*n* = 72), and complex epithelial neoplasms (*n* = 74). Abbreviations: AJCC: American Joint Committee on Cancer. *∗*, local tumor destruction included laser ablation or cryosurgery and electrocautery; fulguration (included the use of hot forceps for tumor destruction). Excision or resection of less than one lobe included excision, laser excision, bronchial sleeve resection only, wedge resection, and segmental resection (including lingulectomy).

**Table 2:** Association between the recommended operation and OS in the crude analysis and multivariable analysis.

Variables	Total	Event (%)	Nonadjusted model		Model 1		Model 2		Model 3	
			HR (95% CI)	*p* value	HR (95% CI)	*p* value	HR (95% CI)	*p* value	HR (95% CI)	*p* value
*Entire cohort*										
Nonoperation	553	494 (89.3)	1 (ref)		1 (ref)		1 (ref)		1 (ref)	
Operation	553	384 (69.4)	0.6 (0.53∼0.69)	<0.001	0.61 (0.53∼0.7)	<0.001	0.61 (0.5∼0.74)	<0.001	0.57 (0.47∼0.69)	<0.001

*Stage IIIA*										
Nonoperation	116	98 (84.5)	1 (ref)		1 (ref)		1 (ref)		1 (ref)	
Operation	85	40 (47.1)	0.44 (0.31∼0.64)	<0.001	0.4 (0.27∼0.59)	<0.001	0.4 (0.19∼0.82)	0.012	0.46 (0.23∼0.95)	0.037

*Stage IIIB*										
Nonoperation	63	56 (88.9)	1 (ref)		1 (ref)		1 (ref)		1 (ref)	
Operation	57	43 (75.4)	0.77 (0.52∼1.15)	0.207	0.82 (0.53∼1.25)	0.358	0.8 (0.41∼1.59)	0.532	0.79 (0.37∼1.69)	0.537

*Stage IIIC*										
Nonoperation	9	8 (88.9)	1 (ref)		1 (ref)					
Operation	11	7 (63.6)	0.48 (0.16∼1.39)	0.174	1.01 (0.27∼3.76)	0.985				

*Stage IVA*										
Nonoperation	349	323 (92.6)	1 (ref)		1 (ref)		1 (ref)		1 (ref)	
Operation	376	278 (73.9)	0.57 (0.49∼0.67)	<0.001	0.58 (0.49∼0.68)	<0.001	0.59 (0.47∼0.75)	<0.001	0.54 (0.42∼0.68)	<0.001

*Stage IVB*										
Nonoperation	16	9 (56.2)	1 (ref)		1 (ref)					
Operation	24	16 (66.7)	1.77 (0.78∼4.03)	0.175	3.39 (1.22∼9.39)	0.019				

Model 1: adjusted for age, sex, race, and marital status at the baseline. Model 2: adjusted for age, sex, race, marital status, primary site, grade, laterality, histology, and AJCC stage. Model 3: adjusted for age, sex, race, marital status, primary site, grade, laterality, histology, AJCC stage, radiation, and chemotherapy.

**Table 3:** The multivariable Cox proportional hazard regression analyses for stage IIIA.

Variables	Unadjusted model		Adjusted model	
	HR (95% CI)	*p* value	HR (95% CI)	*p* value
Nonoperation	Reference		Reference	
Operation	0.44 (0.31∼0.64)	<0.001	0.2 (0.04∼1)	0.051
Age	1.01 (0.99∼1.02)	0.611	1.04 (1∼1.07)	0.058
Sex				
Male	Reference		Reference	
Female	0.54 (0.38∼0.76)	<0.001	0.37 (0.17∼0.82)	0.014
Race				
Caucasian	Reference		Reference	
African American	0.56 (0.24∼1.27)	0.163	0.11 (0.03∼0.39)	0.001
American Indian/Alaska native	NA (NA∼NA)	NA	NA (NA∼NA)	NA
Asian or Pacific Islander	0.82 (0.51∼1.32)	0.408	0.35 (0.14∼0.88)	0.025
Marital status at diagnosis, *n* (%)				
Single	Reference		Reference	
Unmarried or domestic partner	1.6 (0.37∼6.87)	0.524	0 (0∼Inf)	0.998
Married	1.08 (0.66∼1.77)	0.773	0.69 (0.28∼1.73)	0.427
Separated	3.15 (0.42∼23.58)	0.265	NA (NA∼NA)	NA
Divorced	1.34 (0.75∼2.39)	0.328	0.45 (0.13∼1.54)	0.203
Widowed	1.06 (0.62∼1.82)	0.826	0.78 (0.26∼2.31)	0.647
Primary site				
Main bronchus	Reference		Reference	
Upper lobe and lung	0.26 (0.11∼0.66)	0.004	1.4 (0.13∼15.42)	0.784
Middle lobe and lung	0.25 (0.08∼0.76)	0.015	0.72 (0.05∼10.37)	0.807
Lower lobe and lung	0.3 (0.12∼0.77)	0.012	1.29 (0.11∼15.76)	0.841
Overlapping lesion of lung	0 (0∼Inf)	0.995	NA (NA∼NA)	NA
Grade				
I: well differentiated	Reference		Reference	
II: moderately differentiated	3.43 (1.46∼8.09)	0.005	6.2 (2.09∼18.4)	0.001
III: poorly differentiated	4.06 (1.75∼9.4)	0.001	4.68 (1.51∼14.5)	0.008
IV: undifferentiated anaplastic	1.29 (0.27∼6.2)	0.755	0.43 (0.06∼2.96)	0.392
Laterality				
Right	Reference		Reference	
Left	0.99 (0.7∼1.4)	0.957	0.72 (0.37∼1.39)	0.322
Bilateral involvement	1.74 (0.43∼7.11)	0.441	1.05 (0.17∼6.64)	0.959
Histology				
Epithelial neoplasms	Reference		Reference	
Squamous cell neoplasms	0.66 (0.42∼1.03)	0.069	0.21 (0.07∼0.66)	0.008
Adenomas and adenocarcinomas	0.28 (0.18∼0.46)	<0.001	0.12 (0.04∼0.36)	<0.001
Acinar cell neoplasms	0 (0∼Inf)	0.994	0 (0∼Inf)	0.997
Others†	0.23 (0.05∼0.96)	0.044	0.49 (0.07∼3.59)	0.482
Radiation	0.91 (0.65∼1.29)	0.609	1.82 (0.72∼4.59)	0.205
Chemotherapy	0.84 (0.58∼1.2)	0.337	0.44 (0.19∼1.05)	0.064
Type of surgery*∗*				
Nonoperation	Reference		Reference	
Local tumor destruction	1.38 (0.64∼2.98)	0.418	15.3 (1.29∼181.18)	0.031
Resection of less than one lobe	0.4 (0.26∼0.6)	<0.001	2.13 (0.47∼9.64)	0.324
Lobectomy	0.33 (0.12∼0.89)	0.028	NA (NA ∼ NA)	NA
Lobe or bilobectomy extended	NA (NA ∼ NA)	NA	NA (NA ∼ NA)	NA
Pneumonectomy	NA (NA ∼ NA)	NA	NA (NA ∼ NA)	NA
Extended pneumonectomy	NA (NA ∼ NA)	NA	NA (NA ∼ NA)	NA

†, histology and other types included: transitional cell papillomas and carcinomas (*n* = 1), mucoepidermoid neoplasms (*n* = 5), cystic, mucinous, and serous neoplasms (*n* = 72), and complex epithelial neoplasms (*n* = 74). *∗*, local tumor destruction included laser ablation or cryosurgery and electrocautery; fulguration (included the use of hot forceps for tumor destruction). Excision or resection of less than one lobe included excision, laser excision, bronchial sleeve resection only, wedge resection, and segmental resection (including lingulectomy). The model adjusted for age, sex, race, marital status, primary site, grade, laterality, histology, AJCC stage, radiation, chemotherapy, and type of surgery.

**Table 4:** The multivariable Cox proportional hazard regression analyses for stage IVA.

Variable	Unadjusted model		Adjusted model	
	HR (95% CI)	*p* value	HR (95% CI)	*p* value
Nonoperation	Reference		Reference	
Operation	0.57 (0.49∼0.67)	<0.001	0.2 (0.06∼0.65)	0.007
Age	1.01 (1∼1.02)	0.005	1.02 (1.01∼1.03)	<0.001
Sex				
Male	Reference		Reference	
Female	0.78 (0.66∼0.92)	0.003	0.77 (0.61∼0.99)	0.039
Race				
Caucasian	Reference		Reference	
African American	1.1 (0.84∼1.46)	0.488	0.98 (0.62∼1.54)	0.915
American Indian/Alaska native	1.31 (0.54∼3.17)	0.544	2.41 (0.57∼10.15)	0.229
Asian or Pacific Islander	0.59 (0.43∼0.81)	0.001	0.62 (0.4∼0.95)	0.028
Marital status at diagnosis, *n* (%)				
Single	Reference		Reference	
Unmarried or domestic partner	0 (0∼Inf)	0.989	NA (NA∼NA)	NA
Married	1.06 (0.85∼1.33)	0.589	0.74 (0.53∼1.03)	0.076
Separated	0.94 (0.38∼2.32)	0.9	NA (NA∼NA)	NA
Divorced	1.16 (0.86∼1.56)	0.325	0.93 (0.61∼1.41)	0.729
Widowed	1.07 (0.81∼1.41)	0.646	0.71 (0.46∼1.1)	0.129
Primary site				
Main bronchus	Reference		Reference	
Upper lobe and lung	0.67 (0.46∼0.99)	0.043	1.11 (0.62∼2.01)	0.724
Middle lobe and lung	0.58 (0.35∼0.98)	0.043	1.1 (0.53∼2.27)	0.805
Lower lobe and lung	0.71 (0.48∼1.06)	0.097	1.16 (0.64∼2.12)	0.622
Overlapping lesion of lung	0.75 (0.31∼1.8)	0.515	1.65 (0.5∼5.48)	0.414
Grade				
I: well differentiated	Reference		Reference	
II: moderately differentiated	1.09 (0.71∼1.67)	0.707	1.26 (0.79∼2.01)	0.335
III: poorly differentiated	1.2 (0.79∼1.83)	0.384	1.53 (0.97∼2.41)	0.068
IV: undifferentiated anaplastic	1.29 (0.69∼2.42)	0.428	1.97 (1∼3.89)	0.05
Laterality				
Right	Reference		Reference	
Left	0.98 (0.83∼1.16)	0.806	0.95 (0.75∼1.21)	0.684
Bilateral involvement	0.61 (0.37∼1)	0.051	0.99 (0.41∼2.43)	0.99
Histology				
Epithelial neoplasms	Reference		Reference	
Squamous cell neoplasms	1.13 (0.86∼1.49)	0.371	1.09 (0.73∼1.62)	0.685
Adenomas and adenocarcinomas	0.76 (0.6∼0.96)	0.021	0.82 (0.58∼1.17)	0.281
Acinar cell neoplasms	0.47 (0.17∼1.28)	0.139	0.35 (0.11∼1.19)	0.092
Others†	1.07 (0.67∼1.71)	0.775	0.82 (0.41∼1.65)	0.583
Radiation	1.01 (0.85∼1.2)	0.923	1.1 (0.86∼1.41)	0.438
Chemotherapy	0.66 (0.56∼0.77)	<0.001	0.51 (0.4∼0.66)	<0.001
Type of surgery*∗*				
Nonoperation	Reference		Reference	
Local tumor destruction	0.92 (0.67∼1.27)	0.629	5.65 (1.63∼19.61)	0.006
Resection of less than one lobe	0.59 (0.49∼0.71)	<0.001	3.05 (0.93∼10)	0.065
Lobectomy	0.38 (0.28∼0.52)	<0.001	1.49 (0.45∼4.91)	0.512
Lobe or bilobectomy extended	0.25 (0.08∼0.79)	0.018	1.01 (0.2∼5.27)	0.987
Pneumonectomy	0.33 (0.11∼1.03)	0.056	NA (NA ∼ NA)	NA
Extended pneumonectomy	0.58 (0.08∼4.13)	0.586	NA (NA ∼ NA)	NA

†, histology and other types included: transitional cell papillomas and carcinomas (*n* = 1), mucoepidermoid neoplasms (*n* = 5), cystic, mucinous, and serous neoplasms (*n* = 72), and complex epithelial neoplasms (*n* = 74). *∗*, local tumor destruction included laser ablation or cryosurgery, electrocautery; fulguration (included the use of hot forceps for tumor destruction). Excision or resection of less than one lobe included excision, laser excision, bronchial sleeve resection only, wedge resection, and segmental resection (including lingulectomy). The model adjusted for age, sex, race, marital status, primary site, grade, laterality, histology, AJCC stage, radiation, chemotherapy, and type of surgery.

## Data Availability

The data used to support the findings of this study have been deposited in the SEER repository (https://seer.cancer.gov/).

## Supplementary Materials

Supplementary Materials Supplemental Figure 1: SMD plot of each variable after processing using different methods. IPTW: the inverse probability of treatment weighting; SMRW: the standardized mortality ratio weighting; PA: pairwise algorithmic; OW: overlap weight.
