# Supplementary figures and images for: Operative versus Nonoperative Treatment in Patients with Advanced Non-Small-Cell Lung Cancer: Recommended for Surgery
Source: Can Respir J. 2023 Jan 12;2023:4119541. doi: 10.1155/2023/4119541 (PMC9851779; doi:10.1155/2023/4119541)

variable

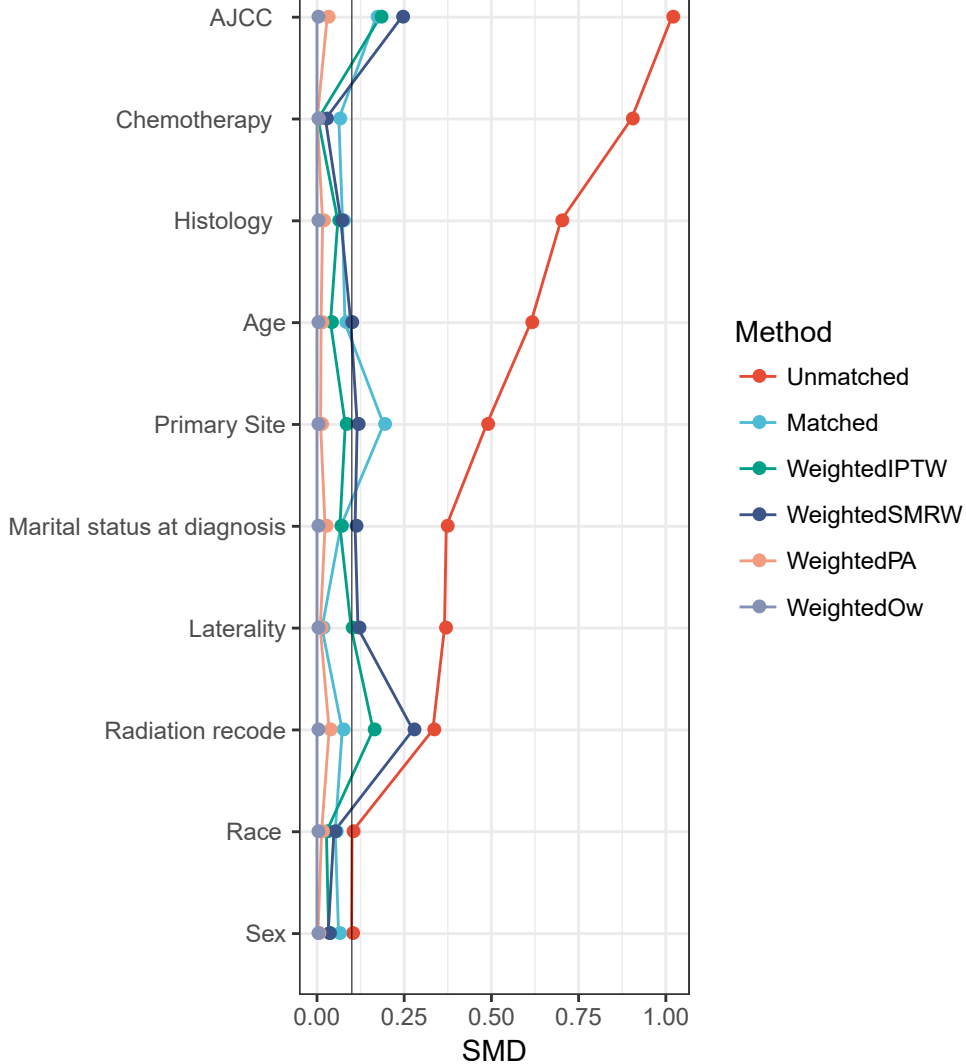

Supplement: Supplementary Materials — Supplemental Figure 1: SMD plot of each variable after processing using different methods. IPTW: the inverse probability of treatment weighting; SMRW: the standardized mortality ratio weighting; PA: pairwise algorithmic; OW: overlap weight. [file 4119541.f1.pdf]
